# Urban Issues: Canyons Up the Pollution Ante

**DOI:** 10.1289/ehp.116-a289a

**Published:** 2008-07

**Authors:** M. Nathaniel Mead

New York City and other mega-cities with populations of 10 million or more are grappling with air pollution associated with traffic congestion, which has been linked with numerous health problems. As part of a larger research effort to understand the costs and benefits of various strategies to reduce traffic congestion in the Big Apple, a new study focuses on how traffic emissions are dispersed within urban street canyons—streets that are lined with tall buildings on both sides.

Within these domains, large quantities of pollutants are released near the ground from motor vehicle exhaust, then trapped and concentrated within the canyon walls. Urban street canyons also tend to contain a lot of people, potentially making these areas high-risk zones for big cities.

“The combination of high population density and high traffic volume in urban areas such as New York City means that the health impact of traffic pollution can potentially be much larger than similar sources in less populated areas,” says Ying Zhou, a research fellow at the Harvard School of Public Health who coauthored a report in the April 2008 issue of *Atmospheric Environment* on urban street canyon modeling in Manhattan. “Street canyons can exacerbate the health impact of traffic pollutants, hence the need to understand their dispersion dynamics.”

Along with colleague Jonathan Levy, an associate professor of environmental health and risk assessment at the Harvard School of Public Health, Zhou has been working on a project to examine how proposed congestion pricing policies in New York City will affect air pollution. Several other cities, including London, Stockholm, Singapore, and three cities in Norway, have implemented congestion pricing systems that charge a fee for vehicles to enter a certain zone in the city, ostensibly to curb traffic in those zones.

“We sought to understand the degree of exposure and public health benefits of control strategies in urban street canyons similar to those in Manhattan, given the hypothesis that the same amount of emissions would have a greater population exposure in a street canyon setting,” says Levy. He and Zhou report that population exposure to traffic pollutants in New York’s urban street canyons can be up to 1,000 times higher than exposure to a similar amount of emissions in other urban settings. Additionally, pedestrians and daytime office workers received most of the population exposure in the midtown Manhattan area, emphasizing the importance of considering nonresidential exposures. In contrast, most studies to date have focused on residential exposures to roadway pollution.

The study focused primarily on the “intake fraction,” a measure of the total population exposure per unit of emissions. “The intake fraction is essentially a function of what individuals are exposed to and how many individuals are exposed,” says Levy. “For example, even though cyclists may be breathing harder than pedestrians, there are many more pedestrians than cyclists passing through urban street canyons in Manhattan, and [pedestrians] move more slowly through the canyon.” Emissions controls in a given street canyon therefore would likely have a larger total health impact on pedestrians than on cyclists, he notes.

The authors speculate that the benefits of pollution controls targeted at urban environments probably have been underestimated because cost–benefit analyses of such controls have not considered either the spatial correlation between high population density and high source density or the dynamic effects of street canyons on pollutant dispersion. The benefits depend on the particular street configuration (with taller buildings and narrower streets amplifying exposures) and on the sizes of various subpopulations (who are affected differentially depending on where they are situated in the street canyon and at what time of day). The benefits per unit emissions are also slightly sensitive to traffic volume, but in an unexpected direction—having more traffic actually decreased the population exposure per unit of emissions (separate from its effect on total emissions) by increasing atmospheric turbulence and pollutant dispersion.

“This study adds to the growing literature on intake fractions for air pollutants and emphasizes that there may be settings that involve relatively small amounts of emissions but contribute substantially to population exposures,” says John Evans, a senior lecturer on environmental science at the Harvard School of Public Health. “Zhou and Levy point out that in urban street canyons mobile source intake fractions may be two to three orders of magnitude larger than in suburban and rural areas. With the rapid growth of mega-cities, urban mobile source emissions become increasingly important determinants of human exposures and health risks. Zhou and Levy’s study makes clear the need for greater emphasis on this issue.”

## Figures and Tables

**Figure:**
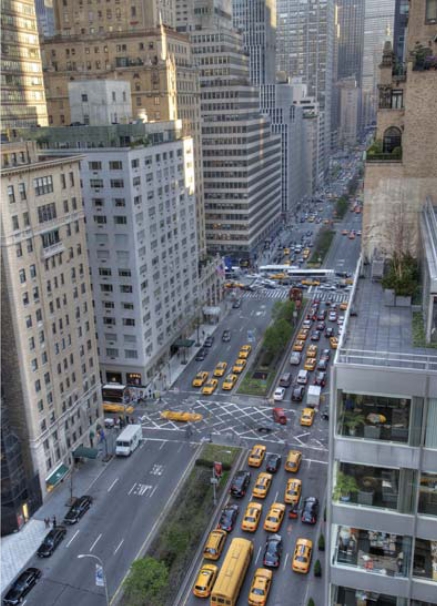
New York City

